# A Modified Technique of Thulium Laser Enucleation for Benign Prostatic Hyperplasia With Non-morcellator Approach

**DOI:** 10.3389/fsurg.2021.657869

**Published:** 2021-05-07

**Authors:** Yifeng Jing, Qian Sun, Wenhuan Guo, Dapeng Zhou, Yiping Zhu, Yuyang Zhao, Di Cui, Xiaohai Wang, Yuan Ruan, Fujun Zhao, Shujie Xia, Bangmin Han

**Affiliations:** ^1^Department of Urology, Shanghai General Hospital, School of Medicine, Shanghai Jiao Tong University, Shanghai, China; ^2^Department of Urology, Tongren Hospital, Shanghai Jiao Tong University School of Medicine, Shanghai, China

**Keywords:** benign prostatic hyperplasia, enucleation, morcellator, thulium laser, modified technique

## Abstract

**Background:** Until recently, most enucleation techniques of the prostate were performed with the application of morcellator. We introduce a modified enucleation technique of thulium laser with non-morcellator approach, which is about incising and vaporizing remaining prostate tissue instead of a morcellator.

**Methods:** A retrospective evaluation of 223 patients undergoing ThuLEP from January 2014 to December 2015 was performed in our institution. One hundred five of the patients used morcellator while the other 118 used non-morcellator approach. All patients were assessed with the International Prostate Symptom Score (IPSS), quality of life (Qol), ultrasonography, serum prostate-specific antigen (PSA), maximal urine flow rate (Qmax), and postvoid residual urine volume (PVR). We reassessed these parameters at 1-, 3-, 6-, and 12-months after operation. Operative time, perioperative, and postoperative complications were also recorded.

**Results:** Significant improvement was noted in the IPSS, QoL, Qmax, and PVR in both groups at the 12-month follow-up, and assessment showed no differences in these parameters between the two groups. Comparisons of the total operation time and enucleation time demonstrated no significant differences between the two groups. Our non-morcellator approach needed more time to incise and vaporize the enucleated tissue compared to morcellation when the prostate volume was about 40–80 ml (*p* < 0.05), while it showed a significant lower rate of superficial bladder injury than using morcellator (*p* < 0.05). There were no significant differences in other complications between the two groups (*p* > 0.05).

**Conclusions:** Our modified technique is a safe and effective procedure for the treatment of BPH avoiding the potential complications caused by morcellator.

## Background

Transurethral resection of the prostate (TURP) is considered as the gold standards for the treatment of benign prostatic hyperplasia(BPH). However, TURP is still associated with significant morbidity including severe bleeding, capsular perforation, and transurethral resection syndrome(TURS) (1). Since the renaissance of laser prostatectomy with the advent of the holmium laser in the 1990s (2), various lasers and subsequent procedures have been introduced (3, 4). Among all kinds of laser-associated techniques, holmium laser transurethral enucleation of the prostate(HoLEP), and thulium laser enucleation of the prostate(ThuLEP) are becoming more and more popular and have been proven to be safe and effective (5–7). Morcellator is widely used in most reported enucleation techniques including HoLEP and ThuLEP. Although morcellation of intravesical adenoma is currently the standard procedure following transurethral enucleation procedures, it can be associated with several morbidities, including ureteral orifice, bladder mucosal injuries even bladder perforation (8, 9). In this study, we introduce a modified ThuLEP without the procedure of morcellation for the treatment of BPH, and make a comparison with ThuLEP with morcellation.

## Methods

### Subjects

From January 2014 to December 2016, a total of 223 symptomatic BPH patients who underwent ThuLEP in our institution were evaluated retrospectively. One hundred five of the patients used morcellator (Group 1) while the other 118 used the new non-morcellator approach (Group 2). Inclusion criteria were as follows: the prostate volume <80 ml, Qmax <15 ml/s, PVR ≥ 50 ml, repeated urinary retention(including patients with indwelling catheterization), medical therapy failure, and lower urinary tract symptoms with IPSS above 7. Patients diagnosed with neurogenic bladder, prostate cancer, or previous prostate surgery were excluded.

All surgeries were performed by two experienced surgeons who had performed over 300 ThuLEP procedures. Perioperative assessment included medical history, digital rectal examination (DRE), IPSS, ultrasonography, PSA, Qmax, QoL, PVR, operative time, catheterization day, resected tissue weight, hemoglobin decrease, hospital stay, and operation-related complications.

### Instruments and Surgical Technique

The instruments used were a 120-W continuous-wave Tm:yttrium aluminum garnet laser (Raykeen), a 26 F continuous flow resectoscope (KARL STORZ) and a mechanical morcellator (Hawk). The laser was used at two different energy levels. The cutting setting was 120 W, and the enucleation setting was 50 W. Laser energy was applied through a reusable 550 lm laser fiber. Physiological saline irrigation was applied throughout the entire procedure.

The technique using morcellator for Group 1 has been previously described in detail (5). The surgical steps of our modified technique of ThuLEP for Group 2 are as follows.

- Making the incision line

**Figure 1:**
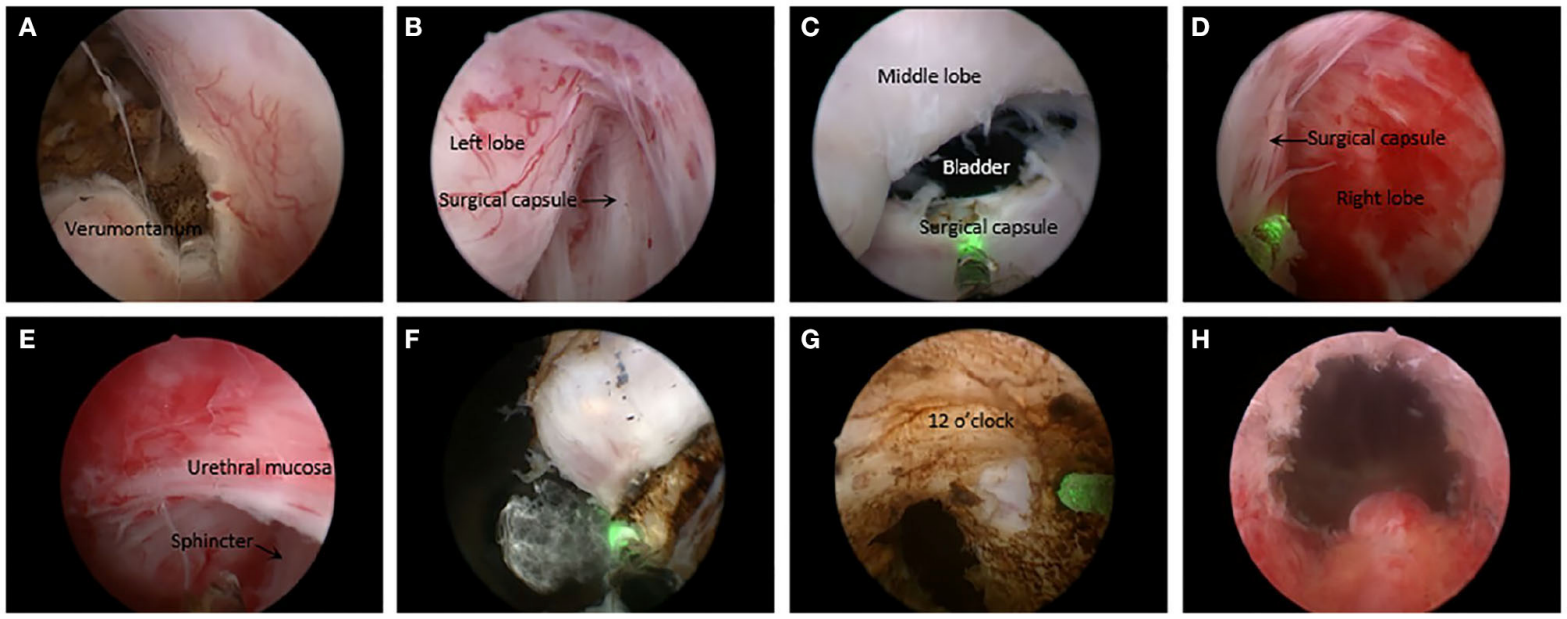
Overview of the non-morcellator technique. **(A)** Making an inverted-U shaped incision line. **(B)** Dissecting the left lobe from the obtained surgical capsule up to 1 o'clock with resectoscope. **(C)** Freeing the middle lobe from the surgical capsule toward the bladder neck. **(D)** Enucleating the right lobe similarly to 11 o'clock. **(E)** Truncating the urethral muscosa along the surface of the enucleated lobe at the apex of prostate. **(F)** Incising and vaporizing the lobes attached to the remaining tissue from 11 to 1 o'clock into small pieces. **(G)** Resecting the remaining tissue from 11 to 1 o'clock. **(H)** Postoperative effect.

An inverted-U-shaped incision around the verumontanum is made. The incision is continued until the surgical capsule is identified (Figure 1A). The laser fiber is then drawn back into the channel of the modified working channel.

- Enucleation

After inspection of the urethral sphincter, the enucleation process goes on along the obtained circumferential surgical capsule from 5 o'clock, the left apex of prostate with the beak of the resectoscope for blunt dissection, freeing the left lateral lobe from the surgical capsule. Then moving it from a counterclockwise fashion along the capsule up to 1 o'clock (Figure 1B), and the left lateral adenoma is gradually released from the capsule with long sweeps from the bladder neck to the apex. Haemostasis is achieved using 50 w laser beam throughout the enucleation as each bleeding vessel is encountered. The middle and the right lateral lobes are similarly enucleated along the capsule from 5 to 11 o'clock (Figures 1C,D). During blunt disconnection, visual control of the surgical capsule and laser coagulation of perforating vessels is necessary. Rather than releasing the lobes completely off the prostatic capsule, we keep the lobes attached to remaining tissue from 11 to 1 o'clock. The way to treat the apex of prostate is truncating the urethral muscosa along the surface of the enucleated lobe (Figure 1E).

- Incision and vaporization

Switching laser energy to 120 W for incision and vaporization, the lobes attached to the remaining tissue from 11 to 1 o'clock is resected into small pieces radially centered on urethra and released to the bladder (Figures 1F,G). Small residual fragments in the bladder are washed out with Ellik. At last, a three-way Foley catheter is placed into the bladder for continuous bladder irrigation with normal saline.

### Statistical Analysis

Normally distributed continuous variables were expressed as the mean ± standard deviation, and they were compared by *t*-tests. Non-normally distributed continuous variables are presented as the median and interquartile range, and they were analyzed with the Wilcoxon rank-sum test. Categorical data were compared by the χ2 test or Fisher's exact test. SPSS 16 software was used for the statistical analysis. A *p* < 0.05 was considered statistically significant.

## Results

Table 1 compares the baseline parameters and primary perioperative outcomes between the 2 groups. There were no significant differences with regard to patients age, prostate volume, IPSS, QoL, Qmax, or PVR (*p* > 0.05).

**Table 1:** Comparison of baseline parameters and primary perioperative outcomes between the 2 groups.

**Parameters**	**Group 1**	**Group 2**	***P*-value**
Number of patients	105	118	
Age (years)	69.2 ± 13.4	67.7 ± 9.9	0.363
Prostate volume (g)	71.2 ± 7.8	69.8 ± 8.4	0.201
IPSS	58.2 ± 21.6	61.5 ± 22.5	0.266
QoL	22.5 ± 5.6	23.5 ± 4.6	0.144
Qmax	4.5 ± 1.1	4.4 ± 0.9	0.456
PVR (ml)	9.3 ± 4.2	8.7 ± 4.1	0.282

*IPSS, International Prostate Symptom Score; QoL, quality of life; Qmax, maximum urinary flow rate; PVR, postvoid residual urine; ThuLEP, thulium laser enucleation of the prostate; TmLRP, thulium laser resection of the prostate*.

The perioperative data are shown in Table 2. Both procedures required a similar operative time, enucleation time, catheterization time, and length of hospitalization. Our non-morcellator approach needed more time to incise and vaporize the enucleated tissue compared to morcellation when the prostate volume was about 40–80 ml (*p* < 0.05), while there were no significant differences between the two groups when it comes to small prostates (<40 ml).

**Table 2:** Perioperative data between 2 groups.

	**Group 1**	**Group 2**	***P*-value**
Operative time (min)	53.8 ± 23.2	56.8 ± 25.6	0.362
Enucleation time (min)	28.4 ± 11.3	27.8 ± 11.8	0.698
Morcellation time (min)/Incising and vaporizing time (min)			
<40 ml	12.5 ± 8.2	13.3 ± 7.4	0.444
40–80 ml	14.2 ± 7.7	18.5 ± 8.7	<0.05
Enucleation/resected tissue weight (g)	61.4 ± 6.6	10.3 ± 7.5	<0.05
Hemoglobin decrease (g/dl)	0.5 ± 0.2	0.5 ± 0. 1	1
Catheterization time (hours)	54.3 ± 4.7	53.3 ± 5.2	0.132
Hospital stay (hours)	58.2 ± 8.3	57.4 ± 8.9	0.488

Table 3 lists the changes in IPSS, QoL, Qmax and PVR at 1, 3, 6, and 12 months. Significant improvements in all these parameters compared with the baseline values were observed at the 1-year follow-up. However, the differences between both groups were not significant.

**Table 3:** Follow-up data for up to 12 months in the two groups.

	**Perioperative**	**Postoperative**				
		**1 month**	**3 months**	**6 months**	**12 months**	***P*2-value**
**IPSS**						
Group 1	22.5 ± 5.6	10.0 ± 6.8	8.1 ± 6.2	7.9 ± 6.2	7.8 ± 5.9	<0.001
Group 2	23.5 ± 4.6	9.4 ± 6.2	8.3 ± 6.5	7.8 ± 6.3	7.7 ± 6.0	<0.001
*P*1 value	0.144	0.392	0.814	0.906	0.903	
**QoL**						
Group 1	4.5 ± 1.1	2.5 ± 1.6	2.1 ± 1.4	1.6 ± 1.5	1.7 ± 1.4	<0.001
Group 2	4.4 ± 0.9	2.3 ± 1.4	2.0 ± 1.6	1.6 ± 1.5	1.9 ± 1.6	<0.001
*P*1 value	0.456	0.321	0.622	1	0.324	
**Qmax**						
Group 1	9.6 ± 4.2	22.6 ± 10.8	23.3 ± 11.3	21.6 ± 9.5	21.3 ± 8.7	<0.001
Group 2	8.7 ± 4.1	23.2 ± 10.4	22.9 ± 11.2	20.8 ± 8.7	22.2 ± 9.5	<0.001
*P*1 value	0.282	0.673	0.791	0.512	0.463	
**PVR**						
Group 1	70.8 ± 83.4	25.2 ± 29.6	24.2 ± 32.3	17.4 ± 30.1	16.9 ± 32.5	<0.001
Group 2	65.8 ± 79.2	23.2 ± 27.8	25.3 ± 30.2	19.4 ± 28.8	18.2 ± 32.7	<0.001
*P*1 value	0.646	0.693	0.793	0.612	0.767	

*P1 value for intergroup comparison. P2 value for the comparison between baseline and postoperative*.

**Figure 2:**
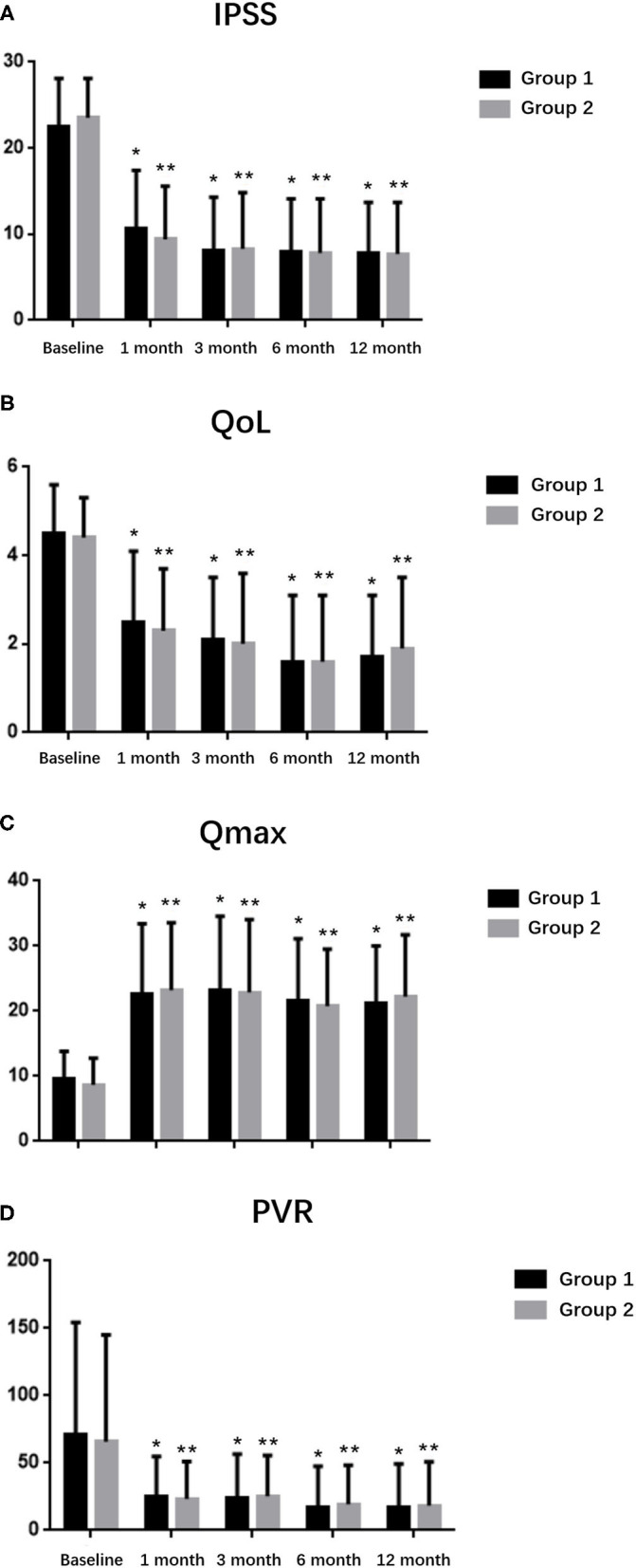
Follow-up data for up to 12 months in the two groups. **(A)** IPSS, **(B)** QoL, **(C)** Qmax, and **(D)** PVR. *P < 0.05 and **P < 0.05 compared with baseline values.

Perioperative complications are listed in Table 4. No cases required second TURP surgery or blood transfusion. There were also no cases of capsular perforation or ureteral orifice injury. Superficial bladder injury occurred in 4 patients in the Group 1, while no patient had this complication in the Group 2. Patients in the Group 1 (4.8%) showed a higher rate than those in the Group 2 (0%), and the difference was significant (*p* < 0.05). Both two groups had 9 patients with urgency urinary incontinence after operation, respectively, but no patients developed urinary incontinence that was persistent for more than 3 months. The incidences of gross haematuria and febrile UTI were not different between the two groups, and they usually resolved with bladder irrigation and antibiotic therapy. The occurrence of Urethral stricture and bladder neck contracture was similar between the two groups, and they were controlled by outpatient urethral dilation. Figure 2 list the changes in IPSS, QoL scores, Qmax and PVR at 1, 3, 6 and 12 months. Significant improvements in all these parameters compared with the baseline values were observed at the 1-year follow-up.

**Table 4:** Adverse events in the 2 groups.

	**Group 1**	**Group 2**	***P*-value**
**Clavien Grade 1 complications**			
Gross hematuria	9 (8.5)	11 (9.3)	0.84
Transitory urge incontinence	9 (8.5)	9 (7.6)	0.80
Temporary urinary retention	5 (4.8)	4 (3.3)	0.60
**Clavien Grade 2 complications**			
Superficial bladder injury	4 (3.8)	0 (0)	0.03
Febrile urinary tract infection (temperature >38.5)	7 (6.6)	6 (5. 1)	0.61
**Clavien Grade 3a complications**			
Bladder neck construction	1 (1.0)	2 (1.7)	0.63
Urethral stricture	2 (1.9)	4 (3.4)	0.49

## Discussion

Laser treatment of BPH through enucleation techniques has become increasingly utilized in the field of urology (10). Similar to open prostatectomy, enucleation of the adenoma carry the advantage of doing an anatomical based deobstruction (11), which may ensure excellent and long-term functional results and low recurrence rate. Multiple competing techniques using different energy sources to accomplish the enucleation procedure have been described, and ThuLEP has been proven to be safe, effective and comparable to HoLEP (11, 12). However, most relevant studies were related with the morcellation procedure, which we believed had some limitations.

Surgeons once used the mushroom method to remove glands after enucleation was performed, which need to substitute electrocautery device during the surgery and it was a very time-consuming process (13). Now with the development of the morcellator (14, 15), surgery for patients with large-sized BPH can be possible (16). However, the smooth surface of the gland and the great mobility of the enucleated pieces made it difficult to catch the targeted pieces. The firmness of the tissue and the dull blades sometimes greatly lowered the suctioning efficiency (17). All these reasons can result in several morbidities, including bladder neck false passage as well as ureteral orifice and bladder mucosal injuries (8, 9, 18). Besides, some researches reported that during the morcellation the bladder is distended to greater than maximal bladder capacity and is likely to result in postoperative voiding difficulties (19).

Thulium laser provides smooth incision and excellent hemostasis with minimal mechanical injury to the pericapsular tissue (20). Given these characteristics, we developed a modified enucleation technique with the use of the Thulium laser instead of morcellator. After the initial attempt, we found the technique was feasible and safe. So we collected the 2-year follow-up information to prove it.

Both groups used the enucleation technique, but the treatment of the enucleated tissue was different. We replaced the morcellation procedure with incision and vaporization of thulium laser. Postoperative functional results showed that PVR, QoL, and IPSS dropped immediately while Qmax increased at first month and this trend continued in the 12 months follow-up period, which confirmed effective anatomic desobstruction and significant relief of symptoms (21).

Compared with Group 1, the operative time in Group 2 was relatively longer, especially when the prostate volume is over 40 ml. That was because the incising and vaporizing time will be prolonged with the increase of prostate volume accordingly. But our data showed the incising and vaporizing time can be similar with morcellation time in condition of small prostate volumes(<40 ml). The catheter inside the urinary bladder depends on postoperative bleeding intensity. The hospital stay and catheterization time were similar with each other, which may be relevant with our careful hemostasis during the surgery. The resected tissue in Group 2 was much smaller than actual resected weight because most tissues were vaporized during the incision procedure.

The incidence of superficial bladder injury was the only postoperative complication that was significantly different in the two groups(*p* < 0.05). For these patients, we need immediate laser hemostasis, prolonged indwelling catheter time, and close observation of urine color and hemoglobin changes. The morcellation procedure has a risk of injury to the bladder mucosa when the bladder is unfilled, sometimes misoperation can also lead to this complication. Relatively, incising and vaporizing the enucleated lobes attached to the remaining tissue from to 1 o'clock is safer than morcellation. Our non-morcellator approach can completely avoid bladder injury.

Our series showed a low incidence of perioperative complications in 223 patients with ThuLEP, which is comparable with other minimally invasive procedures such as HoLEP (6, 22, 23). The mean hemoglobin decrease was 0.5 g/dl and no cases need blood transfusion. No cases of death or secondary surgery also highlight the safety of our methods. Gross hematuria occurs in 9 and 11 cases, respectively, and was resolved spontaneously or with conservative treatment including antibiotic therapy. The incidence of urinary incontinence, including urgency and stress, was up to 8.5 and 7.6%, respectively, when just removing of catheter, which we believed originated from transient urethral dilatation of the apex of the prostate structure. The rate reduced to 2.9 and 2.5% at 1 month and to 0 at 6 month. As reported by Fong et al., avoiding to incising the sphincter base in the anterior area of the prostate during the operation can account for the phenomenon (24).

Several studies indicated a lower incidence of urethral stricture following HoLEP and ThuLEP (25, 26), In our series, the risk of urethral stricture was similar. There were total 6 cases of urethral stricture at first month and 2 cases at third month, we believe it was temporarily associated with urethral edema caused by postoperative inflammation. Only one patient in Group 2 still needed urethra expansion at the twelfth month for urethral stricture that might be related with the patient himself.

Without the morcellation procedure, we avoided the potential occurrence of the superficial bladder mucosal injury and injury to the ureteric orifice.

Some studies reported that plasmakinetic enucleation of the prostate didn't have to use morcellator or change device during the surgery either (27). However, unlike thulium laser incising and vaporizing the tissue accurately by laser fiber, plasmakinetic bipolar resection performed the same procedure with resection loop, which caused smooth wound plane rather than an exact point. When it comes to the apex of prostate, a position needed to be dealt with very carefully in case of urinary incontinence, the thulium laser is supposed to be superior to plasmakinetic bipolar resection, but it need further prospective study.

However, this modified technique had a limitation in that the operation time took longer as the prostate size increased, for the attached tissue is not fixed and may swing to the opposite direction against the fiber. From our experience, if the size of the prostate is too large, the incising and vaporizing procedure can be hard and time-consuming. Thus, it is more suitable for a relatively small prostate. Also, because the data were collected retrospectively, this study could have had a selection bias. However, the results of our study can still be meaningful because of the similar preoperative factors between the groups. Besides, the steep learning curve is a hurdle to overcome for urologists. Finding correct surgical capsule and incising the “half-floating” attached lobes can be difficult and the procedure is better committed to experienced surgeons.

## Conclusions

We introduce a modified technique of ThuLEP without using morcellator. Our initial results prove it was feasible and relatively safe and not inferior to other technique. Further follow-up is needed to prove its long-term durability.

## Data Availability Statement

The original contributions presented in the study are included in the article/supplementary material, further inquiries can be directed to the corresponding author/s.

## Ethics Statement

The studies involving human participants were reviewed and approved by the institutional review board of Shanghai First People's Hospital. The registration number of the trial is ChiCTR1900021072 and the ethical approval number is [2018]61. The patients/participants provided their written informed consent to participate in this study.

## Author Contributions

YJ and QS were responsible for project development, data analysis, data management, and manuscript writing/editing. YJ and BH performed the procedures. YZhu, DZ, DC, XW, YR, and YZha conducted the data collection. WG and FZ performed the data analysis. BH and SX designed the study. All authors read and approved the final manuscript.

## Conflict of Interest

The authors declare that the research was conducted in the absence of any commercial or financial relationships that could be construed as a potential conflict of interest.

## Abbreviations

IPSS: International Prostate Symptom Score
QoL: Quality of life
PSA: Prostate-specific antigen
Qmax: maximal urine flow rate
PVR: Postvoid residual urine volume
TURP: Transurethral resection of the prostate
BPH: Benign prostatic hyperplasia
TURS: Transurethral resection syndrome
HoLEP: Holmium laser transurethral enucleation of the prostate
ThuLEP: Thulium laser enucleation of the prostate
DRE: Digital rectal examination.
